# Semaglutide Has Beneficial Effects on Non-Alcoholic Steatohepatitis in Ldlr-/-.Leiden Mice

**DOI:** 10.3390/ijms24108494

**Published:** 2023-05-09

**Authors:** José A. Inia, Geurt Stokman, Martine C. Morrison, Nicole Worms, Lars Verschuren, Martien P. M. Caspers, Aswin L. Menke, Louis Petitjean, Li Chen, Mathieu Petitjean, J. Wouter Jukema, Hans M. G. Princen, Anita M. van den Hoek

**Affiliations:** 1 Metabolic Health Research, The Netherlands Organization for Applied Scientific Research (TNO), 2333 BE Leiden, The Netherlands; 2 Cardiology, Leiden University Medical Center (LUMC), 2333 ZA Leiden, The Netherlands; 3 Einthoven Laboratory for Experimental Vascular Medicine, Leiden University Medical Center (LUMC), 2300 RC Leiden, The Netherlands; 4 Microbiology and Systems Biology, The Netherlands Organization for Applied Scientific Research (TNO), 2333 BE Leiden, The Netherlands; 5 PharmaNest Inc., Princeton, NJ 08540, USA; 6 Netherlands Heart Institute, 3511 EP Utrecht, The Netherlands

**Keywords:** NAFLD, NASH, inflammation, fibrosis, gene expression

## Abstract

Semaglutide, a glucagon-like peptide-1 receptor agonist, is an antidiabetic medication that has recently been approved for the treatment of obesity as well. Semaglutide is postulated to be a promising candidate for the treatment of non-alcoholic steatohepatitis (NASH). Here, Ldlr-/-.Leiden mice received a fast-food diet (FFD) for 25 weeks, followed by another 12 weeks on FFD with daily subcutaneous injections of semaglutide or vehicle (control). Plasma parameters were evaluated, livers and hearts were examined, and hepatic transcriptome analysis was performed. In the liver, semaglutide significantly reduced macrovesicular steatosis (−74%, *p* < 0.001) and inflammation (−73%, *p* < 0.001) and completely abolished microvesicular steatosis (−100%, *p* < 0.001). Histological and biochemical assessment of hepatic fibrosis showed no significant effects of semaglutide. However, digital pathology revealed significant improvements in the degree of collagen fiber reticulation (−12%, *p* < 0.001). Semaglutide did not affect atherosclerosis relative to controls. Additionally, we compared the transcriptome profile of FFD-fed Ldlr-/-.Leiden mice with a human gene set that differentiates human NASH patients with severe fibrosis from those with mild fibrosis. In FFD-fed Ldlr-/-.Leiden control mice, this gene set was upregulated as well, while semaglutide predominantly reversed this gene expression. Using a translational model with advanced NASH, we demonstrated that semaglutide is a promising candidate with particular potential for the treatment of hepatic steatosis and inflammation, while for the reversal of advanced fibrosis, combinations with other NASH agents may be necessary.

## 1. Introduction

Glucagon-like peptide-1 (GLP-1) receptor agonists are a novel class of agents for the management of hyperglycemia. GLP-1 is an incretin hormone with numerous effects on metabolism, including inhibition of gastric emptying and glucose-dependent stimulation of insulin secretion and promotion of satiety [1,2]. While the beneficial effects of endogenous GLP-1 are limited by its short half-life due to its degradation by dipeptidyl-peptidase-4, GLP-1 receptor agonists are largely protected from degradation and have been shown to be effective in restoring beta cell function [3,4]. Owing to this particular combination of characteristics, GLP-1 receptor agonists provide an effective approach for glucose control that resulted in their approval for management of type II diabetes mellitus [3,4].

Clinical studies have shown that GLP-1 receptor agonists are also effective in lowering body weight, which resulted in their approval for weight management in the context of obesity [5]. Semaglutide and the closely related GLP-1 receptor agonist liraglutide are the first therapeutic agents authorized for the treatment of obesity. Randomized trials using semaglutide demonstrated up to three times greater weight loss compared to other GLP-1 receptor agonists and an on average 25% reduction in energy intake [6,7,8]. Furthermore, clinical studies report that semaglutide is highly effective in improving plasma lipid profiles [9,10,11] and biomarkers of inflammation, including C-reactive protein [9,10,11,12]. Some studies have reported improvement of hepatic function markers; therefore, semaglutide has been proposed for the treatment of non-alcoholic fatty liver disease (NAFLD) and the more severe form non-alcoholic steatohepatitis (NASH) as well [9,13,14]. Current NAFLD-NASH management is mainly focused on lifestyle interventions with the goal of weight loss, whereas the rising prevalence of obesity also necessitates pharmacotherapy.

In this study, we investigated the therapeutic potential of semaglutide as a NASH agent using Ldlr-/-.Leiden mice, which are genetically predisposed to developing cardiovascular disease, obesity, hyperlipidemia and hyperinsulinemia when fed a diet with high levels of saturated fat [15,16,17]. These characteristics develop without requiring extra cholesterol supplementation to the diet. Additionally, Ldlr-/-.Leiden mice develop many pathohistological characteristics of human NASH, recapitulate many changes in gene expression seen in human NASH patients and show significant overlap in underlying disease pathways [17,18,19]. These mice were subjected to a fast-food diet (FFD) for a prolonged period [20,21] to evaluate the effects of semaglutide in a model that closely represents clinically observed metabolic syndrome. The aim of this study was to evaluate the effects of semaglutide in a model with advanced NASH and fibrosis (stage F3) to investigate its therapeutic potential as a NASH agent.

## 2. Results

### 2.1. Semaglutide Improves Metabolic Parameters in Ldlr-/-.Leiden Mice

**Figure 1:**
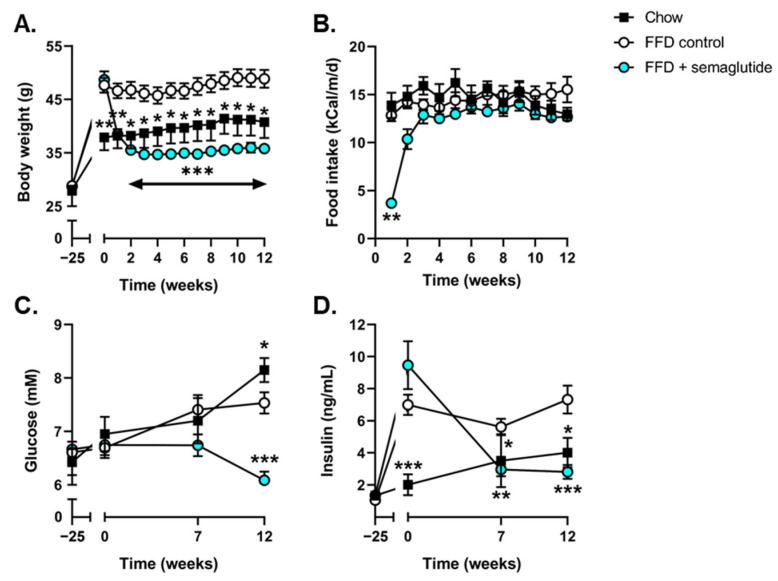
Semaglutide improved metabolic parameters in Ldlr-/-.Leiden mice. Body weight (**A**), food intake (**B**), blood glucose levels (**C**) and plasma insulin levels (**D**) were determined at several timepoints throughout the study. Values are presented as mean ± SEM for n = 8 mice on chow diet, n = 15 on fast-food diet (FFD) control mice and n = 15 mice on FFD supplemented with semaglutide. * *p* < 0.05, ** *p* < 0.01, *** *p* < 0.001 vs. FFD control.

At the start of treatment, Ldlr-/-.Leiden mice had developed pronounced obesity compared with mice fed healthy chow, and their body weight remained stable until the study endpoint (Figure 1A). Twelve weeks of semaglutide treatment significantly reduced body weight (−27%, *p* < 0.001, vs. FFD control at t = 12 weeks) (Figure 1A) despite similar levels of calorie intake (Figure 1B). Suppression of food intake was temporarily observed during the first days of semaglutide intervention; however, food intake was restored within three days to levels comparable to chow and FFD controls. Compared to FFD controls, blood glucose levels in semaglutide-treated mice tended to be decreased after 7 weeks (−9%, *p* = 0.07) and were significantly decreased at t = 12 weeks (−19%, *p* < 0.001) (Figure 1C). Semaglutide significantly reduced plasma insulin concentrations at 7 and 12 weeks after treatment initiation (−47%, *p* < 0.01 and −61%, *p* < 0.001, vs. FFD control at t = 7 and t = 12 weeks, respectively) (Figure 1D).

**Figure 2:**
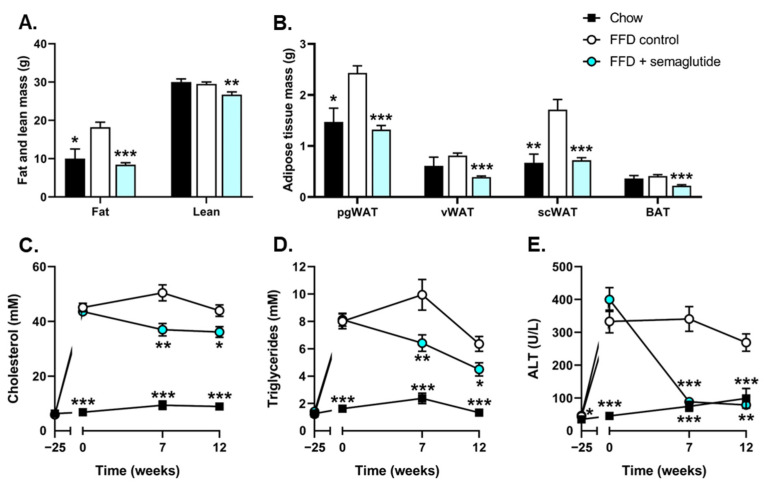
Body composition and plasma levels of cholesterol, triglycerides and ALT were ameliorated in semaglutide-treated mice. Fat mass and lean mass (**A**), perigonadal white adipose tissue (WAT), visceral WAT, subcutaneous WAT and interscapular brown adipose tissue (BAT) (**B**), plasma cholesterol (**C**), plasma triglycerides (**D**) and plasma alanine transaminase (ALT) (**E**) were determined at several timepoints or at the study endpoint. Values are presented as mean ± SEM for n = 8 mice on chow diet, n = 15 fast-food diet (FFD) control mice and n = 15 mice on FFD supplemented with semaglutide. * *p* < 0.05, ** *p* < 0.01, *** *p* < 0.001 vs. FFD control.

Body composition analysis revealed an 82% increase in fat mass in FFD-fed mice (*p* < 0.05) while lean mass remained similar relative to chow-fed mice (Figure 2A). Semaglutide significantly lowered fat mass to levels comparable to those of chow-fed mice (−54%, *p* < 0.001, vs. FFD control) and significantly lowered lean body mass (−9%, *p* < 0.01, vs. FFD control) (Figure 2A). Different adipose tissue depots were weighed at sacrifice and revealed significant increases in perigonadal and subcutaneous white adipose tissue (WAT) weights in FFD control mice compared with chow-fed mice while visceral WAT and interscapular brown adipose tissue (BAT) weights were similar (Figure 2B). Semaglutide significantly lowered the weights of all adipose tissue depots (−45%, −52%, −57%, −47%, all *p* < 0.001, vs. FFD control, for perigonadal, visceral and subcutaneous WAT and BAT, respectively) (Figure 2B).

At t = 0, Ldlr-/-.Leiden mice had developed hypercholesterolemia and hypertriglyceridemia that remained stable during the remainder of the study (cholesterol: 5.6-, 4.4- and 3.9-fold increase, all *p* < 0.001, FFD control vs. chow at t = 0, t = 7 and t = 12 weeks, respectively) (Figure 2C), (triglycerides: 3.9-, 3.2- and 3.8-fold increase, all *p* < 0.001, FFD control vs. chow at t = 0, t = 7 and t = 12 weeks, respectively) (Figure 2D). Semaglutide ameliorated these effects, reflected by significantly lowered plasma cholesterol levels (−27%, *p* < 0.01 and −18%, *p* < 0.05, vs. FFD control at t = 7 and t = 12 weeks, respectively) (Figure 2C) and plasma triglyceride levels (−36%, *p* < 0.01 and −29%, *p* < 0.05, vs. FFD control at t = 7 and t = 12 weeks, respectively) (Figure 2D). FFD feeding increased plasma concentrations of the hepatic function marker alanine aminotransferase (ALT) as well (6.3-fold, *p* < 0.001 at t = 0, 3.6-fold, *p* < 0.001 at t = 7 and 1.7-fold, *p* < 0.01 at t = 12, vs. chow), and semaglutide significantly lowered plasma ALT compared to FFD controls (−74% and −71%, both *p* < 0.001, vs. FFD control at t = 7 and t = 12 weeks, respectively) (Figure 2E), resulting in levels comparable to those of chow-fed mice. 

### 2.2. Treatment with Semaglutide Has Strong Beneficial Effects on Hepatic Steatosis and Inflammation

**Figure 3:**
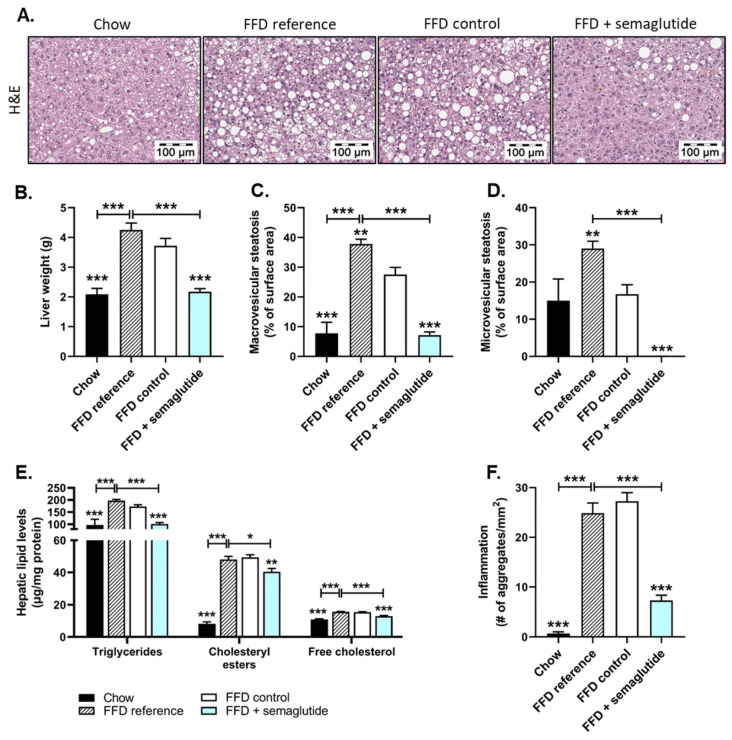
Hepatic steatosis and inflammation were ameliorated with semaglutide treatment in Ldlr-/-.Leiden mice. Representative histological photomicrographs of H&E-stained liver cross-sections (**A**), liver weight (**B**), macrovesicular steatosis (**C**) and microvesicular steatosis (**D**) as percentage of surface area, hepatic concentrations of triglycerides, cholesteryl esters and free cholesterol (**E**) and the number (#) of inflammatory aggregates per mm^2^ microscopic field (**F**) were all determined at the study endpoint. Values are presented as mean ± SEM for n = 8 mice on chow diet, n = 15 on fast-food diet sacrificed at t = 0 weeks, after a 25-week run-in period (FFD reference), n = 15 on FFD until the study endpoint (FFD control) and n = 15 on FFD supplemented with semaglutide. * *p* < 0.05, ** *p* < 0.01, *** *p* < 0.001 vs. FFD control.

At the start of the treatment period at t = 0 (FFD reference) and in the twelve weeks thereafter (FFD control), FFD-fed mice had developed pronounced steatosis (Figure 3A) and significantly increased liver weight (+102% and +77%, for FFD reference at t = 0 and FFD control at t = 12 weeks, respectively, both *p* < 0.001, vs. chow) that was completely reversed by semaglutide treatment (liver weight: −49% and −41%, both *p* < 0.001, vs. FFD reference or FFD control, respectively) (Figure 3B). Histopathological analysis of steatosis revealed that, at the start of the treatment period, two thirds of the liver surface area was steatotic in FFD reference mice, with 38% macrovesicular and 29% microvesicular steatosis (Figure 3C,D). Twelve additional weeks on FFD resulted in a steatotic liver surface area of 28% macrovesicular and 17% microvesicular steatosis. Semaglutide-treated mice showed significant reductions in macrovesicular steatosis (−74%, *p* < 0.001, vs. FFD control) (Figure 3C) to levels comparable to mice on healthy chow. Microvesicular steatosis was completely abolished in semaglutide-treated mice (−100%, *p* < 0.001, vs. FFD control) (Figure 3D), with levels even lower than in chow-fed animals. 

Biochemical analysis of hepatic lipids was consistent with histological analyses and revealed significant increases in triglycerides (FFD reference: +103%; FFD control: +78%, both *p* < 0.001) (Figure 3E), cholesteryl esters (FFD reference: 4.8-fold increase; FFD control: 5.0-fold increase, both *p* < 0.001) (Figure 3E) and free cholesterol (FFD reference: +44%; FFD control: +42%, both *p* < 0.001) (Figure 3E) in FFD-fed mice compared to chow-fed mice. Semaglutide normalized triglyceride content (−41%, *p* < 0.001) and significantly decreased hepatic cholesteryl ester (−18%, *p <* 0.01) and free cholesterol levels (−16%, *p* < 0.001) relative to FFD controls (Figure 3E).

Along with hepatic steatosis markers, the FFD induced hepatic lobular inflammation, characterized by mixed aggregates of mononuclear and polymorphonuclear cells. Contrary to healthy chow-fed mice, FFD reference mice displayed severe hepatic inflammation that persisted in the additional twelve weeks on this diet (FFD control) (Figure 3F). Twelve weeks of semaglutide intervention led to a profound reduction in the number of inflammatory aggregates relative to FFD controls (−73%, *p* < 0.001) and even beyond levels of the start of the treatment (−70%, *p* < 0.001, vs. FFD reference) (Figure 3F).

### 2.3. Semaglutide Does Not Affect Overall Liver Fibrosis Yet Does Improve Collagen Network Architecture

**Figure 4:**
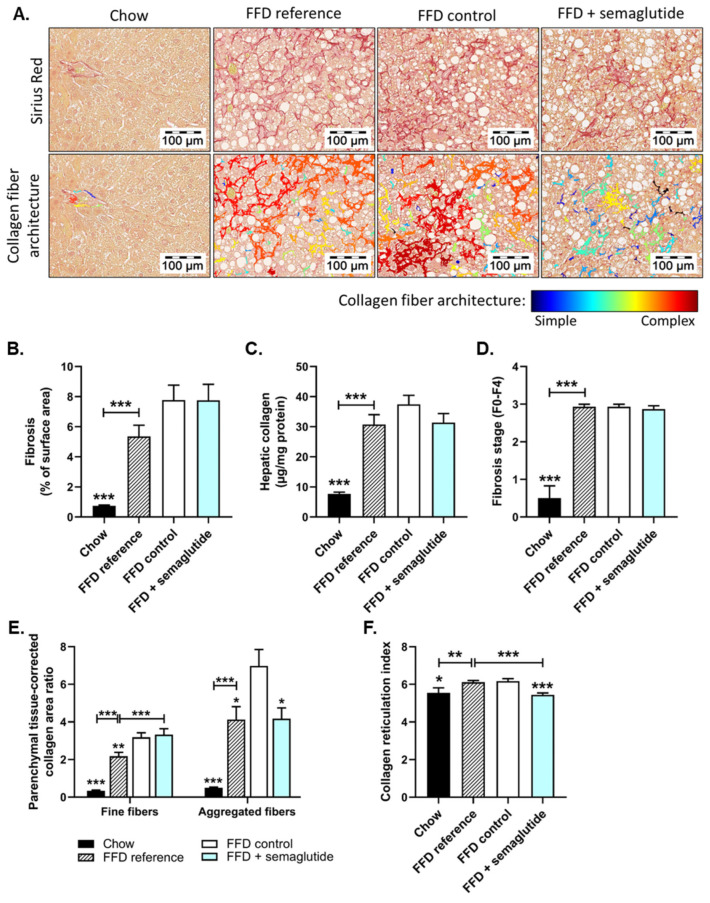
Semaglutide improved hepatic fibrosis architecture in Ldlr-/-.Leiden mice. Representative images of Sirius-Red-stained liver cross-sections and of computerized analysis of collagen fibers of the same section (**A**), fibrosis as percentage of surface area (**B**), hepatic collagen content (**C**), fibrosis stage (**D**), parenchymal-tissue-corrected area ratio of fine and aggregated collagen fibers (**E**) and collagen reticulation index (**F**). Values are presented as mean ± SEM for n = 8 mice on chow diet, n = 15 on fast-food diet sacrificed at t = 0 weeks, after a 25-week run-in period (FFD reference), n = 15 on FFD until the study endpoint (FFD control) and n = 15 on FFD supplemented with semaglutide. * *p* < 0.05, ** *p* < 0.01, *** *p* < 0.001 vs. FFD control.

At the start of the treatment period, the FFD feeding had already induced a considerable amount of fibrosis, reflected by Sirius-Red-stained collagen deposition (FFD reference), which increased further in the twelve weeks thereafter (FFD control) (Figure 4A, upper panels and 4B). This histologically measured collagen deposition was confirmed biochemically by measuring the amount of hepatic hydroxyproline, revealing a 3.0-fold increase for FFD reference mice (*p* < 0.001, vs. chow) and 3.9-fold increase for FFD controls (*p* < 0.001, vs. chow) (Figure 4C). Further evaluation of the fibrosis stage revealed that chow-fed mice either barely developed fibrosis or displayed some fibrosis within perisinusoidal or periportal areas (F0-F1), while mice on FFD developed bridging fibrosis (F3) (Figure 4D). Semaglutide did not affect FFD-induced collagen deposition, measured either histologically or biochemically, nor the fibrosis stage. However, we found a significant positive correlation between the amount of histologically measured fibrosis and the extent of body weight loss in semaglutide-treated mice (r^2^ = 0.495, *p* < 0.01) (Figure S1).

Interestingly, although total fibrosis (collagen % of surface area) was not affected, AI-driven digital fibrosis analysis in Sirius-Red-stained liver sections revealed an amelioration of collagen network architecture with semaglutide treatment (Figure 4A, lower panels). Livers of mice sacrificed after the run-in period (FFD reference) displayed increased area of both fine and thicker/aggregated collagen fibers that continued to increase with twelve additional weeks of FFD feeding (+46%, *p* < 0.01 for fine and +69%, *p* < 0.05 for aggregated collagen fibers, FFD controls vs. FFD reference) (Figure 4E). Compared to FFD controls, semaglutide did not alter the total area of fine collagen fibers yet prevented the increase in total area of aggregated collagen fibers (−40%, *p* < 0.05 vs. FFD control) (Figure 4E). Collagen network complexity was reflected by the collagen reticulation index, which describes the number of intersection points of fine and aggregated collagen fibers in the given area, thereby giving an indication of complexity and related stiffness. As indicated in Figure 4F, the minimal amount of fibrosis in the chow group showed a relatively high degree of collagen network complexity. This index was significantly increased by FFD feeding (FFD reference: +10%, *p* < 0.01; FFD control: +11%, *p* < 0.05 vs. chow) and was lowered by semaglutide intervention (−12%, *p* < 0.001, vs. FFD control) (Figure 4F). Together, these data indicate that while semaglutide does not affect overall collagen content in the liver, it does improve the architectural complexity of the collagen network relative to untreated FFD controls.

### 2.4. Semaglutide Has Minimal Effects on Severe Atherosclerosis

**Figure 5:**
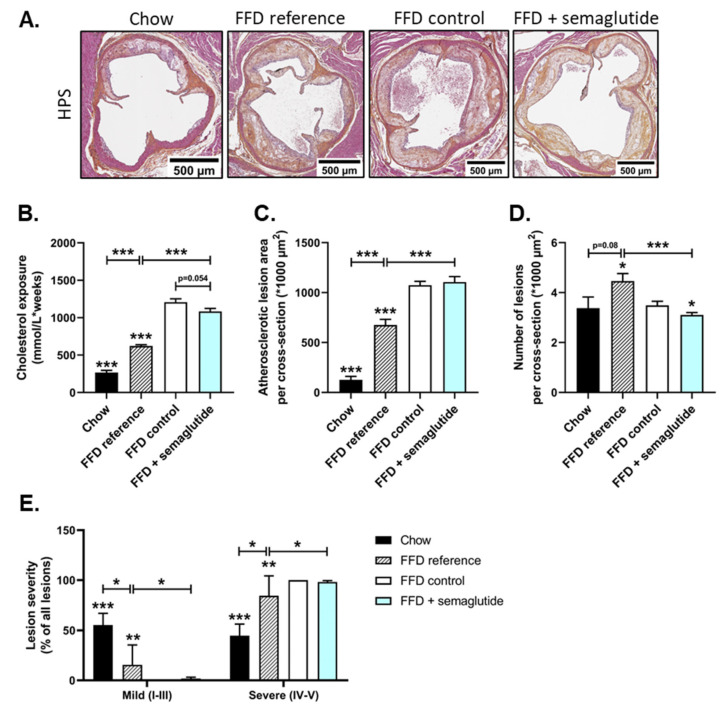
The effects of semaglutide on parameters of atherosclerosis are minimal. Representative images of HPS-stained liver cross-sections (**A**), cholesterol exposure (concentration*weeks) (**B**), atherosclerotic lesion area per cross-section (**C**), number of atherosclerotic lesions per cross-section (**D**) and lesion severity (**E**) were determined at the study endpoint. Values are presented as mean ± SEM for n = 8 mice on chow diet, n = 15 on fast-food diet sacrificed at t = 0 weeks, after a 25-week run-in period (FFD reference), n = 15 on FFD until the study endpoint (FFD control) and n = 15 on FFD supplemented with semaglutide. * *p* < 0.05, ** *p* < 0.01, *** *p* < 0.001 vs. FFD control.

Ldlr-/-.Leiden mice are hyperlipidemic and develop a degree of atherosclerosis during aging on chow, which can be profoundly aggravated by FFD feeding [20]. FFD reference mice had significantly increased total cholesterol exposure (mM*weeks) that was further aggravated by twelve additional weeks on FFD (FFD control) (Figure 5B). Despite significantly lower cholesterol levels in semaglutide-treated mice compared to FFD control mice during the treatment period (Figure 2C), twelve weeks of treatment only tended to lower the total cholesterol exposure during the whole study, including 25 weeks of FFD pre-feeding (−10%, *p* = 0.054). The high cholesterol exposure in FFD-fed groups resulted in the development of severe atherosclerotic plaques in the aortic root (Figure 5A). Quantification of the atherosclerotic lesion area revealed a 4.4-fold increase in FFD reference mice (after 25 weeks of FFD) and a 7.6-fold increase in FFD control mice (after 37 weeks of FFD) relative to chow-fed animals (both *p* < 0.001) (Figure 5C). Twelve weeks of semaglutide treatment did not hamper the progression of atherosclerosis and led to similar lesion areas compared to FFD controls (Figure 5C).

The number of atherosclerotic lesions tended to increase in FFD reference mice (+32%, *p* = 0.08, vs. chow), though this difference was not seen in FFD controls compared to chow-fed mice (Figure 5D). The decreased number of atherosclerotic lesions after an additional twelve weeks on FFD can be attributed to the overall larger plaque size and more severe plaque phenotypes, given that most lesions were severe (type IV or V) (Figure 5E). Although semaglutide intervention led to a significant reduction in the number of atherosclerotic lesions compared to FFD controls (−11%, *p* < 0.05), lesion severity was similar to this group and significantly increased relative to the start of the treatment (FFD reference) (Figure 5D,E). 

### 2.5. Ldlr-/-.Leiden Mice on FFD Closely Represent Human NASH and Semaglutide Improves Hepatic Gene Expression

**Figure 6:**
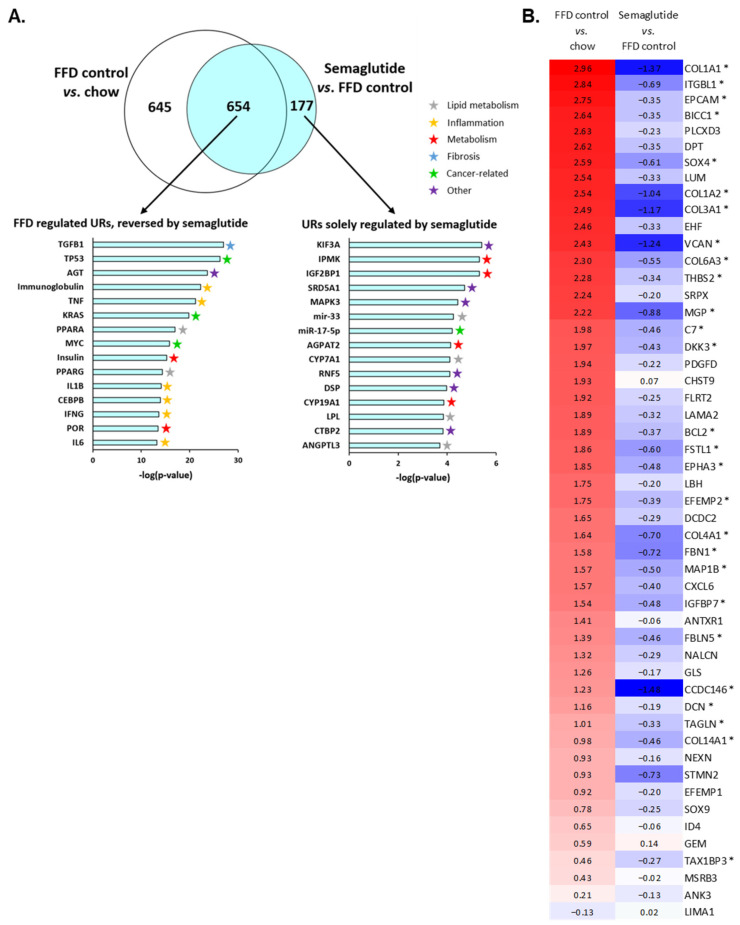
Semaglutide reverses FFD-induced expression of upstream regulators in the Ldlr-/-.Leiden mouse model, which closely represents human NASH. Venn diagram (**A**) showing the overlap of the predicted activation state of upstream regulators (URs) in the liver based on expression changes in known target genes. The white circle indicates Ldlr-/- mice that received the FFD for a total of 37 weeks (FFD control) vs. the healthy chow group, and the blue circle indicates mice treated with semaglutide for the final 12 weeks of the study vs. FFD control mice. The top 15 most significantly changed URs (−log(*p*-value)) for URs affected by FFD and reversed by semaglutide (left) and for URs affected by semaglutide but not induced by FFD (right) are shown. Heatmap (**B**) showing expression of genes differentially regulated in human NASH patients with severe fibrosis (stage F3 or F4) vs. NASH patients with mild fibrosis (stage F0 or F1), recapitulated in Ldlr-/-.Leiden mice fed FFD for a total of 37 weeks (FFD control) relative to chow-fed mice (left column) and semaglutide-treated mice relative to FFD control mice (right column). Red color indicates upregulation, blue color indicates downregulation and asterisks (*) indicate genes that are significantly (*p* < 0.05) upregulated or downregulated in semaglutide-treated mice.

To investigate the mechanistic effects of FFD feeding and semaglutide treatment, we analyzed the hepatic transcriptome and performed an upstream regulator analysis that predicts activation states of proteins, enzymes and transcription factors based on expression changes in downstream genes. Thirty-seven weeks on FFD induced significant enrichment of 1299 upstream regulators compared with healthy chow-fed Ldlr-/-.Leiden mice (Figure 6A, Venn diagram, white circle). Semaglutide treatment resulted in the significant enrichment of 831 upstream regulators (Figure 6A, Venn diagram, blue circle), of which the majority (79%) overlapped with upstream regulators induced in FFD controls. For the overlapping 654 upstream regulators, 96% was either reversed or the effect of the FFD was significantly diminished by semaglutide. Only a small fraction (4%) was enhanced by semaglutide treatment. The top 15 most significantly differentially expressed upstream regulators induced by the FFD and reversed by semaglutide show that most regulators are involved in processes highly relevant to NASH development, including inflammation and lipid metabolism (Figure 6A). The most significantly changed upstream regulator, of which expression was upregulated in FFD controls and reversed by semaglutide, was TGFB1, a principal factor that drives the development of fibrosis. The additional reversed enrichment of inflammation-related genes, including, e.g., TNF, IL1B and IL6, emphasizes the beneficial effects of semaglutide demonstrated at the histological level as well. Moreover, semaglutide caused differential expression of 177 upstream regulators that were not significantly altered in FFD controls. Most of the top 15 upstream regulators solely regulated by semaglutide were involved in lipid or other metabolic processes and were therefore relevant for NASH development as well.

At the histological level, we observed positive effects of semaglutide on collagen network complexity, and transcriptome analysis revealed that TGFB1, an important mediator of fibrosis, was the most significantly enriched upstream regulator due to semaglutide intervention. Therefore, we evaluated the effects of semaglutide on pathophysiological pathways specific for severe fibrosis by comparing the hepatic transcriptomic signature of Ldlr-/-.Leiden mice with a human gene profile published by Moylan et al. that differentiates NASH patients with severe fibrosis (stage F3 or F4) from NASH patients with mild fibrosis (stage F0 or F1) (Figure 6B) [22]. This dataset of genes that were all upregulated in patients with severe fibrosis largely overlapped with the dataset obtained here using next-generation sequencing of liver tissue (Figure S2). The majority of these genes (51 genes) were significantly upregulated in Ldlr-/-.Leiden mice on FFD as well and are shown in Figure 6B, with the exception of LIMA1, which was significantly downregulated. Semaglutide significantly reversed or weakened the expression of at least half of the genes in this dataset compared with FFD controls, while expression of the other half was not significantly modified (Figure 6B). In aggregate, these data demonstrate that the gene profile of mice with FFD-induced NASH shows considerable overlap with patients diagnosed with severe fibrosis and that treatment with semaglutide generally reversed the expression of this gene set.

## 3. Discussion

Semaglutide, a GLP-1 receptor agonist, has been approved for the treatment of type II diabetes mellitus and obesity, yet its effects on the hepatic manifestation of the metabolic syndrome, NAFLD-NASH, are less well recognized. In this study, we used an obese, hyperlipidemic and insulin-resistant mouse model with advanced NASH and hepatic fibrosis to demonstrate that besides its beneficial effects on metabolic parameters, semaglutide improved hepatic steatosis and inflammation and had beneficial effects on collagen network complexity, thereby slightly improving hepatic fibrosis. FFD feeding induced the development of severe atherosclerotic plaques, which was not hampered by twelve weeks of semaglutide treatment. Additional transcriptome analysis and comparison with a human gene profile relevant for human NASH revealed that semaglutide particularly influenced upstream regulators relevant for NASH development and generally reversed hepatic gene expression relevant for severe fibrosis.

Ldlr-/-.Leiden mice were fed a diet rich in saturated fat and fructose without additional cholesterol supplementation to closely mimic the metabolic state of obese human patients [20]. While the predominant carbohydrates in experimental Western-type diets and high-fat diets are glucose and sucrose, the FFD contains almost exclusively fructose [23,24], which results in and more closely recapitulates human NASH [24]. The dietary addition of fructose is particularly relevant, since fructose consumption is presumed to increase NASH severity [25,26]. Here, FFD feeding in Ldlr-/-.Leiden mice induced the development of obesity, hypercholesterolemia, hyperglycemia, hyperinsulinemia and increased plasma ALT concentrations. At the hepatic level, it caused substantial macrovesicular and microvesicular steatosis, along with severe hepatic inflammation. Prolonged exposure to the FFD in the FFD control group relative to the FFD reference group resulted in reduced hepatic macrovesicular and microvesicular steatosis, an aspect of NASH often observed when diets with high fat content are continued over longer periods and that indicates that NAFLD pathogenesis is a highly dynamic process [27,28]. Additionally, the FFD induced the development of severe hepatic fibrosis characterized by a severely reticulated collagen fiber network. These data confirm that the Ldlr-/-.Leiden mouse model closely recapitulates the metabolic state of human NASH patients [20] and can therefore be used to evaluate the efficacy of therapeutic interventions.

In the past decade, several phase II clinical trials have specifically evaluated the efficacy of GLP-1 receptor agonists on treating NAFLD-NASH. These studies confirm the beneficial effects of GLP-1 receptor agonists on glucose, insulin and lipid metabolism [29,30], and others also demonstrate their efficacy on hepatic parameters including plasma ALT, total liver volume and hepatic steatosis [13,14]. Current phase II and phase III trials are investigating the safety and efficacy of semaglutide in NAFLD-NASH patients. In this study, semaglutide treatment was performed at a dosage of 0.12 mg/kg/day. Considering the approximately 12.3 times faster metabolism in mice [31], this would correspond with a dosage of 0.78 mg/day for an 80 kg human or 5.5 mg/week. Despite this dosage being relatively high compared to the clinical semaglutide dosage (2.4 mg/week), it is similar to dosages used in previous preclinical studies in diet-induced obesity models [32,33]. In Ldlr-/-.Leiden mice, semaglutide significantly improved the detrimental effects induced by the FFD. Consistent with clinical studies, semaglutide intervention reduced body weight and shifted body composition to a healthier state with decreased weight of adipose tissue depots [6,14]. Additionally, the suppression of food intake was temporarily observed during the first days of semaglutide intervention, which is consistent with observations in human patients, in whom adverse effects including nausea, diarrhea and reduced appetite are common in the first days after treatment initiation [34]. GLP-1 receptor agonists restore beta cell sensitivity to elevated blood glucose levels and improve overall beta cell function [35], which was reflected here by reductions in blood glucose and plasma insulin levels in semaglutide-treated mice. Improved plasma cholesterol and triglyceride levels in obese patients treated with GLP-1 receptor agonists have been reported in the literature as well [10,12] and are recapitulated in the obese Ldlr-/-.Leiden model.

Besides improving metabolic parameters, semaglutide ameliorated hepatic parameters including plasma ALT levels, which is in line with the clinical data of obese patients [9,13]. Two clinical trials on the effects of semaglutide on NASH parameters both showed significant improvement in hepatic steatosis but not fibrosis [13,14]. Here, we also observed that semaglutide considerably improved hepatic steatosis, owing to significant reductions in macrovesicular steatosis and complete abolishment of microvesicular steatosis. Some clinical studies report improvement of the systemic inflammation marker C-reactive protein due to GLP-1 receptor agonist intervention [36,37]. Nevertheless, little clinical data are available on how they affect hepatic inflammation specifically, often due to the lack of hepatic biopsy and histological confirmation of inflammation. Preclinical studies report positive effects of GLP-1 receptor agonists on hepatic inflammation markers, yet the mechanisms behind these improvements remain incompletely understood [38,39]. In this study, we histologically determined that semaglutide induced a significant reduction in the number of inflammatory cell aggregates in the liver. This positive effect on hepatic inflammation was further substantiated by the transcriptomics analysis, where we found pronounced effects of semaglutide on the expression of upstream regulators involved in inflammatory processes, e.g., TNF, IL1B and IL6. Consistent with clinical studies, semaglutide did not alter the severity of hepatic fibrosis or fibrosis stage induced by FFD feeding. These results are in line with a different preclinical study by Møllerhøj et al., who used C57BL/6J mice fed a diet high in fat, fructose and with 2% cholesterol, where semaglutide improved hepatic steatosis but not fibrosis [33]. In this study, we performed additional AI-driven analysis of collagen fibers, which combines fibrosis traits into a phenotypic fibrosis composite score that is normalized to the parenchymal liver area. By excluding the area affected by hepatic steatosis, this score is corrected for functional tissue. Using this analysis, we have demonstrated that semaglutide does not alter the area ratio of fine collagen fibers yet does prevent the formation of aggregated collagen fibers. In this way, semaglutide-treated mice displayed an overall improvement in collagen reticulation index, a marker of fibrosis complexity.

Although semaglutide shows great potential to reduce hepatic steatosis and inflammation, and we have shown here that it does improve collagen reticulation, it may not be the most suitable for the treatment of advanced, established fibrosis. Our findings are consistent with a recently completed clinical trial in patients with histologically confirmed NASH and stage 4 fibrosis (NCT03987451) that failed to meet its primary endpoint, namely the improvement of liver fibrosis with no worsening of NASH [40]. Although semaglutide improved body weight, plasma ALT levels and hepatic steatosis, liver stiffness determined by magnetic resonance elastography was unchanged compared to placebo-treated NASH patients with NASH-related cirrhosis (stage 4 fibrosis) [40]. In line with our findings, it is suggested that semaglutide is a suitable candidate for the treatment of mild to moderate (F1–F2) fibrosis, while for the treatment of more established fibrosis (F3–F4), combinations with other agents should probably be considered.

Controversy exists about whether GLP-1 receptors are present in the liver, with some studies advocating its presence [41,42] while others refute this notion [43,44,45]. Transcriptomics analysis of livers of Ldlr-/-.Leiden mice on FFD revealed some GLP-1 receptor expression in all mice. From a mechanistic perspective, it is important to understand if the effects of GLP-1 receptor agonists on the liver are direct or indirect. The inconsistent reports on hepatic GLP-1 receptor expression are postulated by Panjwani et al. to be due to a lack of sensitivity and specificity of three commercially available GLP-1 receptor antibodies [45]. These authors found no GLP-1 receptor expression in hepatocytes and suggest that expression detected by others are likely signals originating from, e.g., bile ducts and infiltrating immune cells [45], which is likely the case in the current study as well, since we used whole liver homogenates for transcriptomics analysis. When considering this lack of hepatic GLP-1 receptor expression, the effects of semaglutide on hepatic parameters are likely indirect, e.g., due to reductions in body weight [5]. Gabery et al. included a weight-matched group in their preclinical study and concluded that the beneficial effects of semaglutide could not solely be attributed to weight loss [32]. However, inclusion of a weight-matched group does not mean that these animals have similar body compositions. In our study, we did not include a weight-matched control group yet still saw similar patterns compared to clinical data, where a greater extent of weight loss showed to be associated with histological improvements of NASH parameters [46]. Consistently, we observed that the extent of body weight loss in semaglutide-treated mice correlated with their improvement in hepatic fibrosis (r^2^ = 0.495, *p* < 0.01), indicating that half of the fibrosis reduction may be explained by weight loss.

In this study, we observed minimal effects of semaglutide on atherosclerosis in the current treatment design study. Although cholesterol levels were significantly lower at the study endpoint in semaglutide-treated mice, total cholesterol exposure was still high and not significantly lower than that of FFD controls. The initial 25-week run-in period on FFD induced severe atherosclerosis that worsened even more in the following twelve weeks and that could not be overcome or counteracted by semaglutide. While a different study showed improvement of atherosclerosis parameters in ApoE-/- and Ldlr-/- mice on a Western-type diet with semaglutide treatment [39], the latter study used a prevention design rather than a treatment design. Together, these data indicate that while semaglutide improved metabolic and hepatic parameters, it could not attenuate severe atherosclerosis induced by prolonged exposure to FFD feeding.

By performing transcriptomics analysis of liver tissue, we demonstrated that many upstream regulators showed induced expression by the FFD, and their expression was decreased by semaglutide. Consistent with histological assessment, semaglutide reversed FFD-induced expression of upstream regulators of steatosis and inflammation. One upstream regulator that was induced by FFD feeding and of which expression was most significantly reduced by semaglutide was TGFB1. In the context of NAFLD-NASH, this is of particular interest since TGFB1 drives the development of fibrosis, and we have demonstrated that although fibrosis was not quantitatively affected in this model, remodeling of collagen fibers did take place in semaglutide-treated mice. These results are in line with transcriptomics data reported by a different preclinical study, where it was demonstrated that semaglutide improved expression of several extracellular matrix-associated genes in diet-induced obese mice [33]. Due to the strong effects of semaglutide on TGFB1 and its histologically determined collagen-remodeling properties, we compared the hepatic transcriptome of semaglutide-treated Ldlr-/-.Leiden mice on FFD to a human dataset that differentiates NASH patients with severe fibrosis from NASH patients with mild fibrosis [22]. At the gene expression level, Ldlr-/-.Leiden mice on FFD for a total of 37 weeks closely resembled human NASH patients with severe fibrosis. Intervention with semaglutide generally reversed expression of this gene set, thereby underlining its efficacy on the transcriptome level as well.

In summary, we have demonstrated that FFD-fed Ldlr-/-.Leiden mice respond to semaglutide in a similar fashion as observed in human patients, with resolution of hepatic steatosis and inflammation. Consistent with recent clinical studies, semaglutide did not affect fibrosis quantitatively, yet we observed significant improvement in collagen network complexity. Our findings support the hypothesis that semaglutide is a very promising candidate for treatment of NAFLD-NASH. While semaglutide remains a promising candidate for patients with mild fibrosis (F1–F2), for the treatment of advanced hepatic fibrosis (F3–F4), combinations with additional agents may be considered.

## 4. Materials and Methods

### 4.1. Experimental Design

Animal care and experimental procedures were approved by The Netherlands Central Authority for Scientific Procedures on Animals (CCD; project license AVD5010020172064) and an independent Animal Welfare Body of The Netherlands Organization for Applied Scientific Research (IvD TNO; TNO-489). Ldlr-/-.Leiden mice were bred and housed at the SPF animal facility at TNO (TNO Metabolic Health Research, Leiden, the Netherlands). Male mice were chosen because of their increased susceptibility for developing obesity and inflammation in comparison to female mice [47].

Mice (10–17 weeks old) were group-housed in a temperature-controlled room on a 12 h light–dark cycle at 50–60% humidity and with free access to heat-sterilized water and food. Body weight, food intake per cage and clinical signs were monitored regularly. One group of mice (n = 8) received the healthy grain-based chow diet (Ssniff Spezialdiäten GmbH, Soest, Germany), and a total of 45 mice received fast-food diet (FFD) containing 41 kCal% fat from milk fat, 44 kCal% from carbohydrates (mainly fructose) and 14 kCal% casein (Research Diets, New Brunswick, NJ, USA) to induce advanced NASH and hepatic fibrosis. At t = 0, after a 25-week run-in period on FFD, mice were matched on body weight, glucose, cholesterol and triglycerides into three groups of 15 mice each. Appropriate group sizes were calculated a priori by power analysis (GPower) [48], with a minimal effect size of 30%, using a two-sided test with 95% confidence interval, power of 90% and α of 0.05. One group of n = 15 mice served as FFD reference and was sacrificed at t = 0 to indicate NASH and atherosclerosis severity at the start of the treatment. Comparison of semaglutide-treated mice with this reference group indicated whether semaglutide treatment could reduce NASH, hepatic fibrosis and atherosclerosis below levels at the start of treatment or blocked further progression. The second group (n = 15) received daily subcutaneous saline injections for twelve weeks (FFD control). The third group (n = 15) received daily subcutaneous semaglutide injections (100 µL, Ozempic^®^, Novo Nordisk, Bagsværd, Denmark) for twelve weeks. The dose was escalated over five days at a rate of 0.024 (20%), 0.048 (40%), 0.072 (60%), 0.096 (80%) mg/kg/day until the final dose of 0.120 mg/kg/day was reached and maintained until the study endpoint (based on weekly body weight measurement). Animals were sacrificed non-fasted by gradual-fill CO_2_ asphyxiation after the 25-week run-in period (t = 0; FFD reference) or twelve weeks after the run-in period (t = 12; other groups), and organs were collected for further analysis.

### 4.2. Plasma and Liver Biochemical Analyses

Blood was drawn regularly from the tail vein after a 5 h fasting period into EDTA-coated tubes (Sarstedt, Nümbrecht, Germany). Blood glucose levels were determined at the time of blood sampling using glucose strips and a glucose hand analyzer (FreeStyle Lite, Abbott, Chicago, IL, USA). An enzyme-linked immunosorbent assay (ELISA) was performed to determine plasma insulin levels (#90080; Crystal Chem, Elk Grove Village, IL, USA). Plasma total cholesterol (TC) and triglycerides (TG) were analyzed with enzymatic colorimetric assays (Roche Diagnostics, Almere, the Netherlands). Levels of plasma alanine transaminase (ALT) were determined by reflectance photometry using a Reflotron^®^ Plus analyzer (Hoffman-La Roche, Mannheim, Germany). At the study endpoint (t = 12 weeks), fat and lean mass were determined using an NMR EchoMRI whole-body composition analyzer (EchoMRI 2-in-1, Echo Medical Systems LTD, Houston, TX, USA). Livers, hearts, perigonadal, visceral and subcutaneous white adipose tissue (WAT) and brown adipose tissue (BAT) were collected and weighed. Livers and hearts were formalin-fixed and paraffin-embedded for histological analysis or snap-frozen in liquid nitrogen and stored at −80 °C for biochemical analyses.

Hepatic collagen content was measured in snap-frozen homogenized tissue of the lobus sinister lateralis hepatis with a hydroxyproline-based colorimetric assay using the Sensitive total collagen assay (Quickzyme, Leiden, the Netherlands) and expressed per mg of total liver protein. Concentrations of hepatic TG, free cholesterol (FC) and cholesteryl esters (CE) were determined in snap-frozen homogenized liver tissue (lobus sinister lateralis hepatis) as well, from which lipids had been extracted as described previously [49]. Lipids were separated by high-performance thin-layer chromatography, stained, analyzed with ChemiDoc Touch Imaging System (Bio-Rad Laboratories, Hercules, CA, USA), quantified using Image-Lab version 5.2.1. software (Bio-Rad Laboratories) and expressed per mg of liver protein.

### 4.3. Histological Assessment of NASH

Paraffin-embedded liver tissue of the lobus sinister lateralis hepatis was cross-sectioned (3 µm) and stained with hematoxylin and eosin (H&E) or Sirius Red (SR). A board-certified pathologist blindly scored sections, examining two liver slides per mouse and using an adapted grading system of human NASH [15,50]. Macrovesicular and microvesicular steatosis were determined in two separate cross-sections per mouse at 40× magnification and expressed as percentage relative to the total liver area analyzed. Hepatic inflammation was scored by counting the number of inflammatory cell aggregates per field at 100× magnification (view size 4.2 mm^2^). Five random, non-overlapping fields were examined, and data were expressed as averages of individual scores per mm^2^. For fibrosis scoring, two SR-stained cross-sections of liver tissue per mouse were evaluated with computerized image analysis, and positive staining was expressed as percentage of liver surface area and including blood vessels. Additionally, the pathologist scored fibrosis stage in two cross-sections per mouse using an adapted scoring protocol of Tiniakos et al. [51], in which F0 indicates absence of fibrosis, F1 fibrosis observed within perisinusoidal/perivenular or periportal area, F2 fibrosis within both perisinusoidal and periportal areas, F3 bridging fibrosis and F4 cirrhosis.

AI-driven digital histological analysis of fibrosis was performed using FibroNest^TM^, a single-fiber digital pathology quantitative image analysis and AI platform (PharmaNest, Princeton, NJ, USA) using SR-stained liver sections scanned at 20X on an Aperio AT2 slide scanner (Leica Biosystems, Amsterdam, the Netherlands). After pre-processing steps including color normalization, standardization and segmentation (to eliminate staining variability and artifacts, scanning artifacts, compression artifacts and other sources of noise), each collagen fiber was identified and segmented as an individual object using a combination of specialized thresholding and AI methods. To account for the evolution of collagen fibers from faint and simple fibers to more complex and networked structures, fibers were classified into fine and assembled fibers based on the number of skeleton nodes of their individual skeletons. In the same sections used for AI-driven measurements of fibrosis, steatosis was evaluated. Fine and assembled collagen fiber scores were normalized to the parenchymal liver area of the same section. By excluding the area affected by macrovesicular steatosis, the fibrosis score was corrected for functional tissue. In addition, the architecture of the collagen phenotype was quantified using the normalized ratio of skeleton branch lengths to the total lengths of skeletons (collagen reticulation index). This measure informs on the structure and interconnection of the collagen network and accordingly provides an estimation on the complexity of hepatic fibrosis.

### 4.4. Histological Assessment of Atherosclerosis

Atherosclerosis was histologically assessed as previously described [52,53,54]. In short, hearts were fixed in formalin at sacrifice, embedded in paraffin and sectioned perpendicular to the axis of the aorta. Cross-sections (5 µm) at 50 µm intervals were hematoxylin–phloxin–saffron (HPS) stained and scanned using an Aperio AT2 slide scanner (Leica Biosystems, Amsterdam, the Netherlands). Per mouse, four sections at 50 µm intervals were assessed for atherosclerotic lesions, and total lesion area per cross-section was calculated. For determination of severity, lesions were classified into five categories in accordance with the American Heart Association’s classification where I indicates early fatty streak, II regular fatty streak, III mild plaque, IV moderate plaque and V severe plaque [52,53].

### 4.5. Transcriptome Analysis

Next-generation sequencing was carried out as described previously [18]. In short, the RNA-Bee total-RNA isolation kit (Bio-Connect, Huissen, the Netherlands) was used to isolate total RNA from individual liver samples (lobus sinister lateralis), and RNA integrity was determined with the RNA 6000 Nano Lab-on-a-Chip kit and bioanalyzer 2100 (Agilent Technologies, Amstelveen, the Netherlands). Total RNA was processed into tagged random sequence libraries (NEBNext Ultra II Directional RNA Library Prep Kit, NEB #E7760S/L for Illumina, Biolabs; including fragmentation, cDNA synthesis, tagging by ligation with sample-specific adapters, PCR amplification) and quality checked for proper size distribution (300–500 bp peak, Fragment Analyzer) by service provider GenomeScan B.V. (Leiden, the Netherlands). The mixed (multiplex) sample libraries were sequenced (NovaSeq6000 v1.5, Illumina, San Diego, CA, USA) with a paired-read 150-cycle sequencing protocol (~20 million reads/sample). The resulting sequence reads (fastq files) were quality-trimmed and paired (trimmomatic), mapped (STAR) to the mouse reference genome (Mus_musculus.GRCm38.gencode.vM19: fasta-sequence and gtf-annotation file) and counted (htseq), resulting in a raw count matrix with # of reads per gene (rows) and sample (columns). No outlier samples were identified (outlier if within treatment group > between treatment group variance in PCA or clustering of normalized DESeq2 counts). Differentially expressed genes were identified using the Deseq2 method [55]. The Ingenuity Pathway Analysis (IPA, Ingenuity Systems Inc., Redwood City, CA, USA, www.ingenuity.com, accessed 17 March 2022) program was used to identify the effects of semaglutide on activity of upstream regulators, which include transcription factors as well as receptors, metabolites and enzymes. This program predicts activation or deactivation of upstream regulators based on gene expression data where z-scores > 2 indicate enhanced activity and z-scores < −2 indicate reduced activity of upstream regulators [18]. The dataset of this study is accessible at the NCBI Gene Expression Omnibus (GEO) database with accession number GEO226496. Additionally, the effects of semaglutide on pathophysiological pathways specific for severe fibrosis were evaluated by comparing the gene expression in Ldlr-/-.Leiden mice with published data of a study that distinguishes NASH patients with severe fibrosis (stage F3 or 4) from NASH patients with mild fibrosis (stage F0 or F1) (GEO accession number GSE31803) [22].

### 4.6. Statistical Analysis

Data are presented as mean ± standard error of the mean (SEM) and differences between groups were determined non-parametrically by Kruskal–Wallis testing followed by Mann–Whitney U testing for independent samples. SPSS software (version 25, ICM Corp., Armonk, NY, USA) was used for all statistical analyses. Two-tailed *p*-values are reported, and a *p*-value ≤ 0.05 was considered statistically significant. The correlation between body weight loss and histologically scored fibrosis was calculated with a Spearman’s rank-order correlation test. For transcriptome analysis, we selected differentially expressed genes using *p*-values adjusted for multiple testing (False Discovery Rate: FDR < 0.001). Upstream regulators were selected based on *p*-values of Fischer’s exact test in the Ingenuity Pathway Analysis software (QIAGEN IPA Winter Release (December 2021) (*p*-value/pathway < 0.01).

## Data Availability

The data presented in this study are available in this article and the accompanying Supplementary Material. The mouse gene expression dataset used for transcriptomics analysis is publicly available via Gene Expression Omnibus, accession number GEO226496.

## Supplementary Materials

The supporting information can be downloaded at: https://www.mdpi.com/article/10.3390/ijms24108494/s1.
