# Supplementary material for: Semaglutide Has Beneficial Effects on Non-Alcoholic Steatohepatitis in Ldlr-/-.Leiden Mice
Source: Int J Mol Sci. 2023 May 9;24(10):8494. doi: 10.3390/ijms24108494 (PMC10218334; doi:10.3390/ijms24108494)
Supplement: Supplementary file 1 [file ijms-24-08494-s001.zip › ijms-2390289-supplementary.pdf]

International Journal of
Molecular Sciences

Supplementary Material

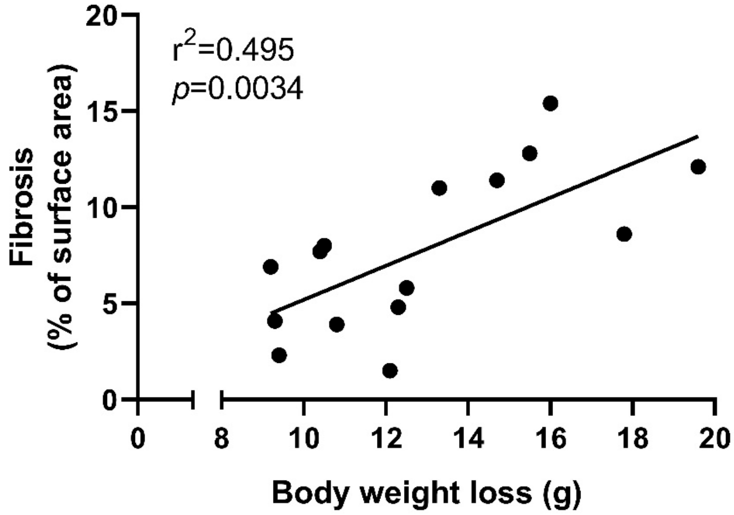

Figure S1. Correlation between body weight loss and fibrosis as percentage in semaglutide-treated mice. At the study endpoint, body weight loss of each individual mouse was plotted against histologically scored fibrosis expressed as percentage of surface area. A Spearman's rank-order correlation test was performed for n=15 mice in the semaglutide-treated group.

FFD control vs. chow	Semaglutide vs. FFD control	
2.96	-1.37	COL1A1 *
2.84	-0.69	ITGBL1 *
2.75	-0.35	EPCAM *
2.64	-0.35	BICC1 *
2.63	-0.23	PLCXD3
2.62	-0.35	DPT
2.59	-0.61	SOX4 *
2.54	-0.33	LUM
2.54	-1.04	COL1A2 *
2.49	-1.17	COL3A1 *
2.46	-0.33	EHF
2.43	-1.24	VCAN *
2.30	-0.55	COL6A3 *
2.28	-0.34	THBS2 *
2.24	-0.20	SRPX
2.22	-0.88	MGP *
1.98	-0.46	C7 *
1.97	-0.43	DKK3 *
1.94	-0.22	PDGFD
1.93	0.07	CHST9
1.92	-0.25	FLRT2
1.89	-0.32	LAMA2
1.89	-0.37	BCL2 *
1.86	-0.60	FSTL1 *
1.85	-0.48	EPHA3 *
1.75	-0.20	LBH
1.75	-0.39	EFEMP2 *
1.65	-0.29	DCDC2
1.64	-0.70	COL4A1 *

(continued)

1.58	-0.72	FBN1 *
1.57	-0.50	MAP1B *
1.57	-0.40	CXCL6
1.54	-0.48	IGFBP7 *
1.41	-0.06	ANTXR1
1.39	-0.46	FBLN5 *
1.32	-0.29	NALCN
1.26	-0.17	GLS
1.23	-1.48	CCDC146 *
1.16	-0.19	DCN *
1.01	-0.33	TAGLN *
0.98	-0.46	COL14A1 *
0.93	-0.16	NEXN
0.93	-0.73	STMN2
0.92	-0.20	EFEMP1
0.86 (n.s.)	0.34	CLDN11
0.78	-0.25	SOX9
0.65	-0.06	ID4
0.59	0.14	GEM
0.46	-0.27	TAX1BP3 *
0.43	-0.02	MSRB3
0.37 (n.s.)	0.43	Fxyd2
0.25 (n.s.)	-0.36	GLT8D2
0.23 (n.s.)	-0.36	JAG1 *
0.21	-0.13	ANK3
0.09 (n.s.)	0.29	AQP1 *
0.08 (n.s.)	-0.41	PNMA1
-0.10 (n.s.)	0.00	C1orf198
-0.11 (n.s.)	-0.03	CYBRD1
-0.13	0.02	LIMA1
-0.61 (n.s.)	0.30	CLDN10

Figure S2. All genes significantly upregulated in NASH patients with severe fibrosis versus NASH patients with mild fibrosis. Heatmap showing expression of all genes differentially regulated in human NASH patients with severe fibrosis (stage F3 or F4) *vs.* NASH patients with mild fibrosis (stage F0 or F1), recapitulated in Ldlr-/-.Leiden mice fed FFD for a total of 37 weeks (FFD control) relative to chow-fed mice (left column) and semaglutide-treated mice relative to FFD control mice (right column). All genes in the human dataset are shown, genes that were not significantly upregulated or downregulated in the FFD control group are indicated with n.s. (not significant). Red color indicates upregulation, blue color indicates downregulation and asterisks (*) indicate genes that are significantly ($p<0.05$) upregulated or downregulated in semaglutide-treated mice.
